# Supplementary material for: Spinal Astrocyte-Neuron Lactate Shuttle Contributes to the Pituitary Adenylate Cyclase-Activating Polypeptide/PAC1 Receptor-Induced Nociceptive Behaviors in Mice
Source: Biomolecules. 2022 Dec 12;12(12):1859. doi: 10.3390/biom12121859 (PMC9775268; doi:10.3390/biom12121859)
Supplement: Supplementary file 1 [file biomolecules-12-01859-s001.zip › biomolecules-2044651-supplementary proofed.pdf]

Supplementary Materials

Spinal Astrocyte-Neuron Lactate Shuttle Contributes to the Pituitary Adenylate Cyclase-Activating Polypeptide/PAC1 Receptor-Induced Nociceptive Behaviors in Mice

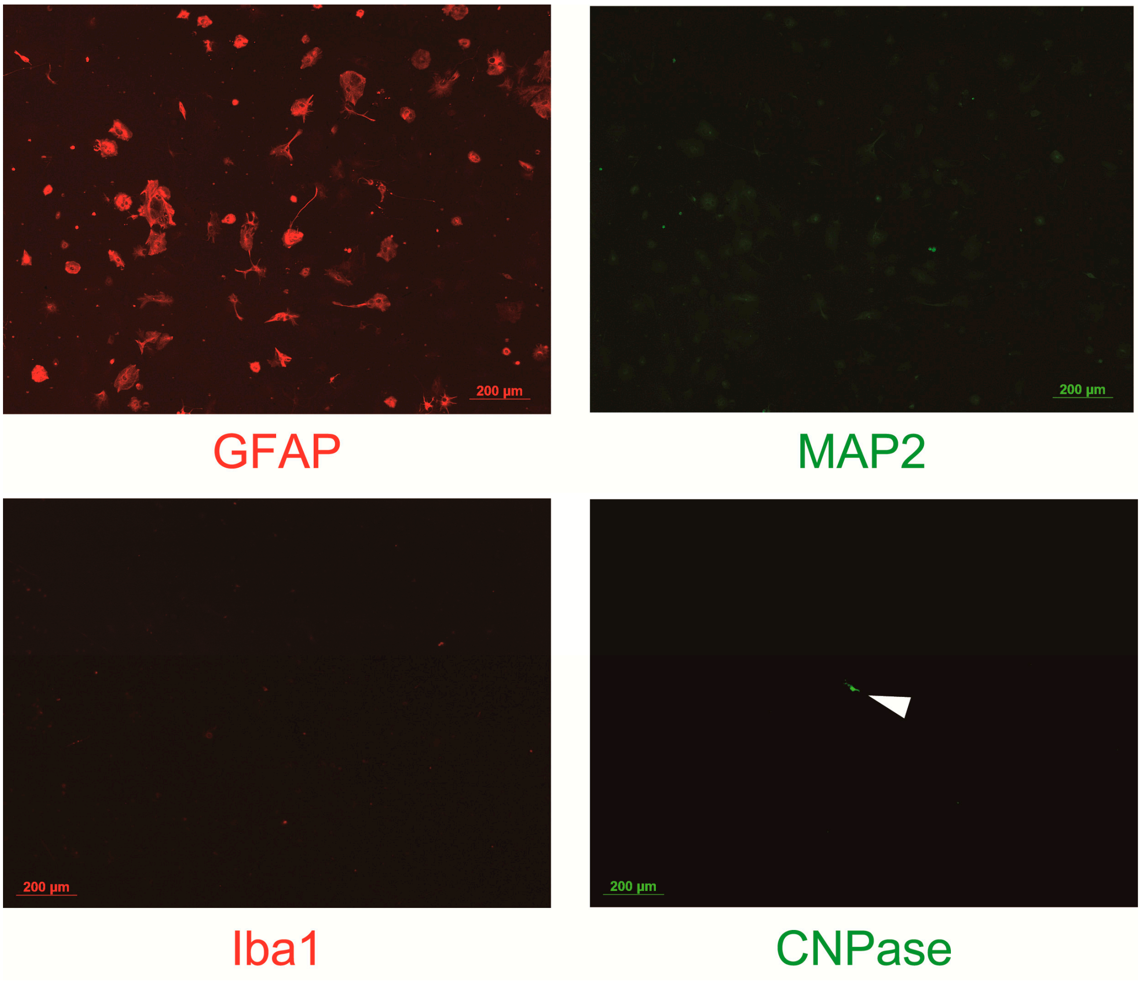

Figure S1. Representative immunofluorescent images of the cultured spinal astrocytes. The purity of astrocytes was examined by staining with primary antibodies against GFAP (an astrocyte marker), MAP2 (a neuron marker), Iba 1 (a microglia marker) and CNPase (an oligodendrocyte marker). Each marker was visualized by an appropriate fluorescent

dye-conjugated secondary antibody. Arrowhead represented an incidentally contaminated oligodendrocyte. Bar = 200 μm.

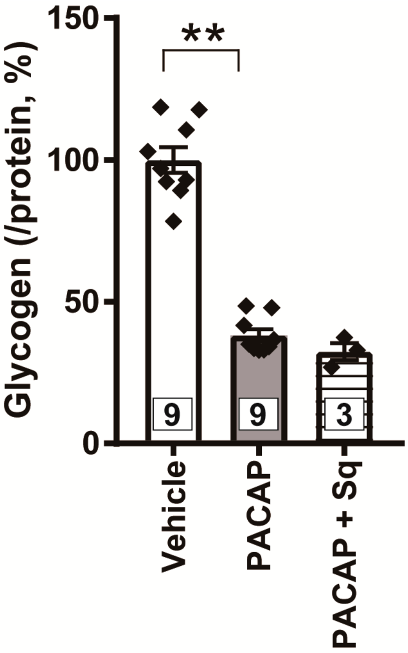

Figure S2. PACAP/PAC1 receptor-evoked glycogenolysis might not be mediated by G_s protein/PKA pathway in the cultured spinal astrocytes. Cultured spinal astrocytes were pre-incubated with Sq22.536 (Sq, 100 μM) for 30 min prior to PACAP (1 nM) exposure, and glycogen amounts contained in the astrocytes were measured 60 min after the PACAP exposure. **P<0.01 when compared with Vehicle. Statistical significance was evaluated by the Dunnett test. Exact sample sizes are indicated in the graphs.
